# West Nile Virus Outbreak in North American Owls, Ontario, 2002

**DOI:** 10.3201/eid1012.040167

**Published:** 2004-12

**Authors:** Ady Y. Gancz, Ian K. Barker, Robbin Lindsay, Antonia Dibernardo, Katherine McKeever, Bruce Hunter

**Affiliations:** * University of Guelph, Guelph, Ontario, Canada;; † Health Canada, Winnipeg, Manitoba, Canada;; ‡ The Owl Foundation, Vineland, Ontario, Canada

**Keywords:** West Nile virus, epidemiology, owl, bird, Icosta americana, Hippoboscidae, Ontario, Canada, research

## Abstract

From July to September 2002, an outbreak of West Nile virus (WNV) caused a high number of deaths in captive owls at the Owl Foundation, Vineland, Ontario, Canada. Peak death rates occurred in mid-August, and the epidemiologic curve resembled that of corvids in the surrounding Niagara region. The outbreak occurred in the midst of a louse fly (*Icosta americana*, family *Hippoboscidae*) infestation. Of the flies tested, 16 (88.9 %) of 18 contained WNV RNA. Species with northern native breeding range and birds >1 year of age were at significantly higher risk for WNV-related deaths. Species with northern native breeding range and of medium-to-large body size were at significantly higher risk for exposure to WNV. Taxonomic relations (at the subfamily level) did not significantly affect exposure to WNV or WNV-related deaths. Northern native breeding range and medium-to-large body size were associated with earlier death within the outbreak period. Of the survivors, 69 (75.8 %) of 91 were seropositive for WNV.

## Abstract 2

Susceptibility of North American owls to WNV is associated with native breeding range.

Since its initial detection in the New York City area in 1999 (*1*), West Nile virus (WNV) has emerged as a health risk for humans and has been associated with illness and death in a wide variety of North American birds, mammals, and reptiles (*2**,**3*). In addition, serologic evidence of exposure to this arthropodborne flavivirus (family *Flaviviridae*) has been reported in many other North American species in which disease had not been previously recorded (*4**–**6*).

While severe clinical disease develops in a minority of humans and horses with WNV infection (*7**,**8*), the situation in birds appears to be different in North America. Some North American birds are highly susceptible to this virus, as shown by large-scale local deaths of American Crows (*Corvus brachyrhynchos*, family *Corvidae*) (*1**,**9*). This finding was further demonstrated recently by experimental infection (*10*). The factors that make some species highly susceptible to WNV remain largely unknown.

Studying the effect of taxonomic, geographic, and demographic background on susceptibility to WNV is potentially useful for predicting and modeling the effect of WNV on host populations. So far, taxonomy alone has offered limited help in predicting susceptibility to WNV, as some closely related species (e.g., within the same genus) show different susceptibility patterns. For example, Fish Crows (*C. ossifragus*) appear less susceptible to WNV than do American Crows (*10*).

Geographic distribution could explain susceptibility patterns if a species has had previous exposure to the same or similar agent. Exposure to St. Louis encephalitis virus (SLEV) has been suggested as a possible mechanism of acquired immunity against WNV (*2*). In addition, species that have evolved in areas where other flaviviruses, such as SLEV, are common may have undergone selection for an innate immune response that may offer protection against WNV.

Age-related differences in susceptibility to WNV have been described for chickens (*2*) and domesticated geese (*11*). These age-related differences have not been reported in wild birds, perhaps because of the difficulty determining their age.

From July to September 2002, high death rates occurred in captive owls (family *Strigidae*) kept at the Owl Foundation, Vineland, Ontario, Canada. At the time, many of the birds were infested with adult hematophagous louse flies (order Diptera, family *Hippoboscidae*); some had loads >400 flies per bird. Initially, the deaths were attributed to this infestation. On August 9, the authors examined three dead owls. Necropsy findings included marked hepatomegaly, splenomegaly, and cerebral hemorrhage. A rapid antigen-capture assay for WNV detection (Vec Test, Medical Analysis Systems, Camarillo, CA) was used for initial screening of oropharyngeal swabs from these birds; however, all samples were negative. On August 16, tissue samples from eight owls, including the initial three, were found to be positive for WNV by reverse transcription–polymerase chain reaction (RT-PCR) test.

Beginning August 23, the remaining birds were vaccinated with a killed WNV vaccine (West Nile Innovator Vaccine, Fort Dodge Animal Health, Fort Dodge, IA). However, from July 26 to September 28, a total of 108 (44%) of 245 owls died.

To our knowledge, the outbreak at the foundation is the largest WNV outbreak in captive wildlife collections in North America since 1999 (*12**–**14*) and the first in Canada. These outbreaks offer a unique and transient opportunity to study the effect and epidemiologic features of WNV infection in multiple species under quasinatural conditions. With the implementation of preventative measures against WNV (e.g., vector control and vaccination), this opportunity will disappear.

The objective of this study was to describe the epidemiologic features of the WNV outbreak at the foundation in 2002. Specifically, we studied the effect of outdoor housing, age, body size, taxonomy, and native breeding range on exposure to WNV and on WNV-related deaths.

## Materials and Methods

### Study Site

The Owl Foundation specializes in breeding and rehabilitating North American owls. Its facility in the Niagara region (Vineland, Ontario; 43°10´ N, 79°20´ W) has ≈3,340 m^2^ of specially designed outdoor cages and a few indoor cages.

### Records and Observations

The Owl Foundation maintains detailed records of all birds in the facility, which includes each bird's history, date admitted, species, cage in which it is housed, movements, and medical history. These data were used in the epidemiologic analysis of this outbreak. For most birds in this study, sex had not been determined.

Data summarizing dead corvid sightings in the Niagara region (1,896 km^2^ in size, map available at http://www.regional.niagara.on.ca/exploring/pdf/regional-niagara.pdf) were obtained from the Canadian Cooperative Wildlife Health Center national WNV surveillance database. These data were gathered by the Niagara Region Health Unit from May 14 to October 12, 2002.

## Sample Collection

Complete diagnostic necropsies were performed at the Ontario Veterinary College on 94 owls and one falcon (family *Falconidae*) that died at the Owl Foundation from April 15 to December 25, 2002. This broad time frame was to facilitate detection of the first and last WNV-related deaths in the 2002 outbreak. All carcasses were kept frozen at –20°C from shortly after death and were allowed to thaw at 4°C for 24 to 48 h before examination (March–August 2003). For each bird, samples of brain, lung, liver, spleen, and kidney were pooled and refrozen at –70°C until analyzed by real time RT-PCR. Small core samples of brain, kidney, and liver were collected with a 20-gauge spinal needle from three additional carcasses that had not been available for necropsy.

During the outbreak, several hundred louse flies were collected at the foundation and frozen at –20°C. These flies were collected off sick or dead birds, as well as from birds that appeared healthy. Six flies were submitted for species identification, while whole-body homogenates of 23 flies, including 18 that were removed from dead or sick owls, were tested for WNV by real time RT-PCR.

From January 22 to May 1, 2003, a serologic survey of all outbreak survivors was conducted. Blood samples were collected from the jugular or cutaneus ulnar vein and placed in heparinized tubes. Plasma was then separated by centrifugation and frozen at –70°C until analyzed.

### Real-time RT-PCR

Real-time RT-PCR assay was used to detect WNV RNA as previously described (*15*). Samples consisted of homogenates prepared from pooled brain, lung, liver, spleen, and kidney (approximately 1 mm^3^ of each tissue). RNA was extracted by using the RNeasy 96 viral isolation kit (Qiagen, Inc.,Valencia, CA). The ABI Prism 7700 Sequence Detection System and the TaqMan One Step PCR Master Reagents Kit (PE Applied Biosystems, Foster City, CA) were used for the assay. Positive controls consisted of three 10-fold dilutions of Egypt 101 (Eg 101) strain of WNV. Three sets of negative (water) controls were used, two during the extraction procedure and one during amplification (i.e., no template). Extracts were screened with the generic 3´ NC primer set. Positive samples underwent a second RNA extraction and were then tested with both the 3´ NC and WNV primer sets. Primer sequences were as described (*15*). Samples that had threshold cycle <37 with both primer sets were considered positive. Homogenates prepared from whole louse flies were tested with the same procedures described for owl tissues.

### Serologic Testing

We used an enzyme-linked immunosorbent assay (ELISA) recently shown to detect avian anti-WNV immunoglobulin (Ig) G in 23 avian species of 12 orders, including a Barred Owl (*Strix varia*) (*6*). The assay was performed as described, with slight modifications. Briefly, the inner 60 wells of a 96-well plate were coated with the monoclonal antiflavivirus antibody 4G2. After the wells were treated with blocking buffer, WNV recombinant COS-1 viral antigen (Centers for Disease Control and Prevention, Atlanta, GA) and a control recombinant COS-1 antigen (Centers for Disease Control and Prevention) diluted to 1:100 were added to the top and bottom 48 wells of the plate, respectively. Plasma samples diluted 1:400 were added, as were positive and negative controls. Horseradish peroxidase-conjugated goat anti–wild bird IgG (Bethyl Laboratories, Inc., Montgomery, TX) was added and allowed to bind to anti-WNV antibodies before the substrate tetramethylbenzidine (Kirkegaard & Perry Laboratories, Inc., Gaithersburg, MD) was added. The reaction was stopped after 30 min by H_2_SO_4_ and read by a microtiter plate reader at 450 nm. Samples showing positive and negative optical density ratios >2 at the 1:400 dilution were considered positive.

A subset of 20 plasma samples representing eight species of owls and four species of raptors was tested by plaque reduction neutralization tests (PRNT). The assay was performed as described (*4*). Titers were expressed as the highest dilution that produced >90% reduction in plaque number. Titers >40 were considered positive. The controls used were back titrations of WNV (NY strain) at 2.5 x 10^7^ PFU/mL diluted to 100, 10, and 1 PFU and a negative control (media only). PRNT assays were performed in a biosafety level 3 facility at the National Microbiology Laboratory, Winnipeg, Manitoba.

### Study Population

The period between the first and last WNV-related deaths at the Owl Foundation was determined from real-time RT-PCR results and defined as the West Nile outbreak period. The study population included 245 birds that were at the foundation on the first day of the outbreak period. These birds represented 16 species of North American owls, one species of Eurasian owl, and two species of falcons (Table 1). Most were permanently disabled birds of wild origin; some had spent many years at the Owl Foundation, while others were recent additions or hatchlings. Ten birds were housed indoors, and 235 were kept outdoors.

**Table 1:** Deaths at the Owl Foundation property during a WNV outbreak (July 26–September 28, 2002) and results of real-time RT-PCR on tissues from dead birds^a^

Species	At risk	Died	Tested	Positive n (%)	Crude DR %	WNV-related DR %
Snowy Owl (*Bubo scandiacus*)	20	20	11	11 (100)	100	100
Northern Hawk Owl (*Surnia ulula*)	26	26	17	17 (100)	100	100
Great Gray Owl (*Strix nebulosa*)	27	27	23	21 (91.3)	100	91.3
Boreal Owl (*Aegolius funereus*)	11	11	11	10 (90.9)	100	90.9
Northern Saw-whet Owl (*Ae. acadicus*)	13	12	12	12 (100)	92.3	92.3
Northern Pygmy Owl (*Glaucidium gnoma*)	6	1	1	1 (100)	16.7	16.7
Short-eared Owl (*Asio flammeus*)	16	2	2	2 (100)	12.5	12.5
Flammulated Owl (*Otus flammeolus*)	9	1	1	1 (100)	11.1	11.1
Long-eared Owl (*A. otus*)	13	3	3	1 (33.3)	23.1	7.7
Great Horned Owl (*B. virginianus*)	22	2	1	1 (100)	9.1	4.5
Barn Owl (*Tyto alba*)	10	0	0	–	0	0
Barred Owl (*S. varia*)	8	1	1	0 (0)	12.5	0
Burrowing Owl (*Athene cunicularia*)	10	0	0	–	0	0
Eastern Screech Owl (*Megascops asio*)	36	0	0	–	0	0
Elf Owl (*Micrathene whitneyi*)	1	0	0	–	–	–
Spotted Owl (*S. oocidentalis*)	1	1	1	1 (100)	–	–
Tawny Owl (*S. aluco*)	2	1	1	1 (100)	–	–
American Kestrel (*Falco sparverius*)	2	0	0	–	–	–
Peregrine Falcon (*F. peregrinus*)	2	0	0	–	–	–
Total	235	108	85	79 (92.9)	46	43

^a^WNV, West Nile virus; RT-PCR, reverse transcription–polymerase chain reaction; DR, death rate (calculated when n > 6).

### Statistical Analysis

Logistic regression was used to test the effect of outdoor placement on WNV-related deaths. Once this effect was established, birds kept indoors were excluded from further analysis. Logistic regression was then used to test the effect of taxonomy, native breeding range, age, and species body size on exposure to WNV and on WNV-related deaths (among exposed birds). Species were considered northern if their reported native breeding range was largely north of latitude 48°N or "other" if it was not so (*16**–**19*). Affiliation (at the subfamily level) followed previously published taxonomic classifications (*20*). Species were further classified as small, medium, or large if their average body weight was <250 g, 250–500 g, or >500 g, respectively (*20*). The two age groups compared were <1 year and >1 year. A general linear model was used to test the effect of the same factors on the date of death (i.e., the number of days to death from when the index bird died). Regression procedures were performed using the SAS software (version 8.2, SAS Institute Inc., Cary, NC). When the odds ratio (OR) could not be calculated directly because of a zero value, an approximation of the OR was calculated by adding 0.5 to each cell (*21*). For the purpose of this analysis, eight moribund birds that were euthanized during the outbreak were considered to have died spontaneously. Seven birds that died during the outbreak but were negative for WNV, were excluded from all statistical analysis. The κ statistic (Quickcalcs, GraphPad Software, Inc., San Diego, CA) was used to test for agreement between ELISA and PRNT results.

## Results

**Figure 1:**
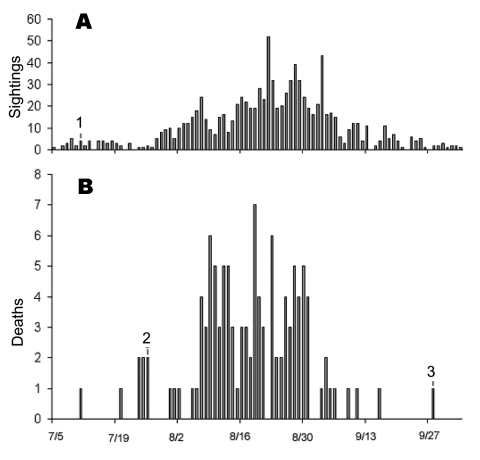
A) Dead corvid (family *Corvidae*) sightings at the Niagara region, July 5–October 4, 2002, and B) daily death rates at the Owl Foundation during the same period. 1) First WNV-positive crow, 2) first and 3) last WNV-related deaths at The Owl Foundation are shown.

A total of 138 birds died in the Owl Foundation from April 15 through December 30, 2002. Thirteen of these died due to known causes other than WNV, leaving 125 that died of unknown cause. Of these, 98 were tested by real-time RT-PCR for WNV, and 80 (81.6%) were positive. Based on these results, the outbreak period was July 26–September 28, 2002. The first five cases occurred during an 11-day period in five different cage complexes scattered throughout the facility. Further analysis of spatial patterns was not attempted because the birds frequently moved between cages during the outbreak period. Daily deaths at the Owl Foundation and dead corvid sightings in the Niagara region from July 5 through October 4, 2002, are shown in Figure 1. Within the outbreak period, 79 (92.9 %) of 85 birds that died were positive for WNV based on RT-PCR. Table 1 summarizes the results of birds tested by species and shows species-specific death rates.

A total of 91 outbreak survivors, which were kept outdoors during the entire outbreak period and not vaccinated against WNV, were tested by ELISA for anti-WNV IgG. Of these, 69 (75.8%) were seropositive. Agreement between ELISA and PRNT results was good with κ = 0.857 (0.58–1.13). The two tests produced conflicting results for 1 of 20 samples; ELISA results are shown in Table 2. Species-specific exposure rates are shown in Table 3. The overall exposure rate was 84.3%.

**Table 2:** Results of enzyme-linked immunosorbent assay for detection of anti-WNV IgG in survivors^a^ of a WNV outbreak, the Owl Foundation, 2002^b^

Species	Tested	No. seropositive (%)
Barn Owl (*Tyto alba*)	10	8 (80)
Short-eared Owl (*Asio flammeus*)	8	8 (100)
Barred Owl (*Strix varia*)	2	2 (100)
Great Horned Owl (*Bubo virginianus*)	12	12 (100)
Burrowing Owl (*Athene cunicularia*)	10	9 (90)
Flammulated Owl (*Otus flammeolus*)	8	1 (12.5)
Eastern Screech Owl (*Megascops asio*)	33	24 (72.7)
Northern Pygmy Owl (*Glaucidium gnoma*)	5	2 (40)
Elf Owl (*Micrathene whitneyi*)	1	1 (100)
Peregrine Falcon (*Falco peregrinus*)	2	2 (100)
Total	91	69 (75.8)

^a^Birds kept outdoors and not vaccinated against WNV. ^b^WNV, West Nile virus; Ig, immunoglobulin.

**Table 3:** Exposure^a^ rates and WNV-related death rates for 12 species of owls kept outdoors at the Owl Foundation property during WNV outbreak, 2002^b^

Species^c^	Exposure rate (%)	WNV-related DR (%)
Snowy Owl (*Bubo scandiacus*)	100	100
Northern Hawk Owl (*Surnia ulula*)	100	100
Northern Saw-whet Owl (*Aegolius acadicus*)	92.3	92.3
Great Gray Owl (*Strix nebulosa*)	100	91.3
Boreal Owl (*Ae. funereus*)	90.9	90.9
Northern Pygmy Owl (*Glaucidium gnoma*)	50	16.7
Short-eared Owl (*Asio flammeus*)	100	12.5
Flammulated Owl (*Otus flammeolus*)	22.2	11.1
Great Horned Owl (*B. virginianus*)	100	4.5
Burrowing Owl (*Athene cunicularia*)	90	0
Eastern Screech Owl (*Megascops asio*)	72.7	0
Barn Owl (*Tyto alba*)	80	0
All species	84.3	43

^a^Exposure defined as positive reverse transcription–polymerase chain reaction result (for birds that died during the outbreak) (Table 1) or positive serologic results (for birds that survived the outbreak) (Table 2). ^b^WNV, West Nile virus; DR, death rate. ^c^Only species for which n > 6 are shown.

Being kept outdoors during the outbreak period was found to be a highly significant risk factor (p < 0.0001) for WNV-related death. None of the 10 birds kept indoors died during the outbreak period, despite the fact that 8 of 10 belonged to species that otherwise had very high death rates (four Northern Hawk Owls, three Boreal Owls, and one Northern Saw-whet Owl). These birds were excluded from further analysis.

Species' northern native breeding range and large-to-medium body size were significant risk factors for exposure to WNV (p < 0.05) with OR = 52.56 (95% confidence interval [CI] 3.13–881.84) and OR = 16.82 (95% CI 3.79–74.67), respectively. Age and taxonomy at the subfamily level were not significant risk factors for exposure. Among exposed birds, northern native breeding range was a highly significant risk factor for WNV-related death (p < 0.0001), with OR = 1,507 (95% CI 85.51–26,557). Birds >1 year of age were also more likely to die of WNV (p < 0.05). with OR = 4.87 (95% CI 2.46–9.62). Size and subfamily were not found to be significant risk factors for WNV-related death.

Native breeding range was significantly associated with the date of death (p < 0.01). Northern species died earlier during the outbreak period (mean and standard deviation 23.8 ± 9.8 days, n = 93) compared to other species (35.0 ± 12.9 days, n = 8). Large or medium birds also died earlier during the outbreak period (21.9 ± 8.5 days, n = 77) compared to small species (33.9 ± 11.2 days, n = 24) (p < 0.05). Subfamily and age were not significantly associated with the date of death.

The six louse flies examined were identified as *Icosta americana* (order Diptera, family *Hippoboscidae*). WNV RNA was detected by real-time RT-PCR in 16 (88.9%) of 18 flies collected from dead or sick owls during the outbreak period. Five adult flies collected from birds that appeared healthy were WNV negative.

## Discussion

WNV RNA was present in most owls that died at the Owl Foundation during the outbreak period, and antibodies against WNV were detected in most outbreak survivors (overall exposure rates 84.3%). The similarity between the epidemiologic curve of the outbreak at the Owl Foundation and that of dead corvid sightings in the Niagara region (Figure 1) and the fact that the initial cases occurred in several cages scattered throughout the Owl Foundation property, suggest that the outbreak was part of a regional WNV activity rather than a point-source introduction (e.g., by admitting a viremic bird). As none of the birds kept indoors were affected, the major route of WNV transmission likely was vector-borne.

Northern native breeding range and large-to-medium body size were significant risk factors for exposure to WNV, while age and subfamily were not. Birds that attract more vectors have a higher risk for exposure to WNV, and the host's body size may be an important determinant of vector attraction, as reported for mosquitoes and sandflies (*22**–**24*). Why species' breeding range should affect exposure is less obvious. However, northern species may attract more feather-dwelling arthropods, such as louse flies, because of their thick feathering.

WNV RNA was detectable in 88.9% of *I. americana* louse flies collected off dead or sick birds. If this parasite can transmit WNV, the overall high exposure rates seen at the Owl Foundation would be explained in light of the louse fly infestation. Subjective observations made by the Owl Foundation staff suggest that northern owl species had the heaviest infestation. If confirmed, these findings could explain why northern species had higher exposure rates; however, this subject requires further investigation.

Looking at the species-specific death rates (Table 1), the distribution of WNV-related deaths was uneven. Given the high overall ER, this finding suggests marked differences in species susceptibility to the virus, with species falling into one of three groups (for scientific names see Table 1): death rates >90% (Snowy Owl, Great Gray Owl, Northern Hawk Owl, Boreal Owl, and Northern Saw-whet Owl), death rates <20% (Long-eared Owl, Short-eared Owl, Great Horned Owl, Flammulated Owl, and Northern Pygmy Owl), and death rates = 0% (Barn Owl, Burrowing Owl, and Eastern Screech Owl).

Affiliation at the subfamily level did not significantly affect death rates. Susceptibility to WNV-related death crossed taxonomic lines and was strongly related to native breeding range. This finding together with the serologic data suggest that death rates were not determined by the ability of WNV to infect different owl species but rather by the ability of each species to survive the infection. All owl species showed either serologic or pathologic evidence of WNV infection.

Immunity to WNV, as for other pathogens, can be either innate or acquired. If immunity to WNV in this case was acquired, one could expect that northern owl species that spent years at the Owl Foundation would be just as resistant as the locally breeding Eastern Screech Owls. Furthermore, if this supposition was the case, young age should have been a significant risk factor, as juvenile birds regardless of species would have been less likely to have acquired immunity. Our data show the opposite; birds >1 year were at a significantly higher risk for WNV-related death. The reason for this finding is unclear and may suggest that juvenile owls have some resistance to WNV infection.

**Figure 2:**
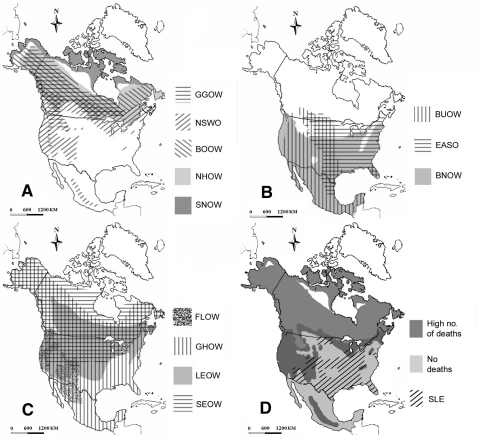
A) Native breeding range of owl species showing high death rates (>90%), B) no deaths, C) low death rates (<20%), and D) the combined distributions of species in the high and no mortality groups with that of Saint Louis encephalitis (SLE) in the United States and Canada. The distribution maps have been redrawn based on maps previously published (*16**–**19*). The distribution of SLE is based on human cases reported in the United States and in Canada from 1964 to 2000 (*25**,**26*). Only states and provinces that had >1 case per 100,000 capita during this period were included. GGOW, Great Gray Owl; NSWO, Northern Saw-whet Owl; BOOW, Boreal Owl; NHOW, Northern Hawk Owl; SNOW, Snowy Owl; BUOW, Burrowing Owl; EASO, Eastern Screech Owl; BNOW, Barn Owl; FLOW, Flammulated Owl; GHOW, Great Horned Owl; LEOW, Long-eared Owl; SEOW, Short-eared Owl.

Innate immunity could have evolved through selection if some of the species coexisted for long periods of time with agents similar to WNV. Based on their native breeding range, northern owl species may have little or no exposure to SLEV (Figure 2). Indeed, species in the high (>90%) death rates group are all northern species, most of which breed north of latitude 48°N, with some exceptions. Great Gray and Boreal Owls have southern extensions to this range, which follow the distribution of high-altitude conifer forest. The Northern Saw-whet Owl has a complex distribution, but all Northern Saw-whet Owls at the Owl Foundation have likely originated in the species' boreal population. Species that showed no WNV-related deaths have relatively southerly distributions, while species in the low (<20%) death rate group have intermediate or very widespread native breeding range (Figure 2).

The link between native breeding range and susceptibility to WNV is intriguing. A similar relation between northern distribution and susceptibility to aspergillosis has been documented in a variety of avian species with northern native breeding range, including owls (*27*). T-cell–mediated immunity is essential for fighting fungal pathogens (*28*) and is also believed to play an important role in the immune response against flaviviruses (*29**,**30*). Northern species may have a less effective cell-mediated immune response to pathogens that are scarce or nonexistent in their natural environment.

Northern native breeding range and large-to-medium body size were significantly associated with earlier death during the outbreak period. This finding could be a result of infection at an earlier date, shorter incubation, shorter disease course, or a combination. Northern owl species had high death rates and may have had a more acute form of the disease. Larger birds, by attracting more vectors, may have been infected earlier in the outbreak period or received a higher dose of WNV.

While concluding that the differences observed in WNV-related death rates are the result of differential innate immunity of the various owl species, and that this pattern follows a north-south distribution based on host-pathogen coevolution is tempting, this finding does not appear to be the case for other North American species. American Crows, for example, have a wide native breeding range (*16**–**19*) and yet appear to be very susceptible to WNV (*1**,**9**,**10*). In addition, contributing factors cannot be ruled out. The outbreak occurred during an exceptionally dry and hot summer concurrently with an *I. americana* infestation. These conditions may have been especially harsh on the northern species, likely causing stress and immune suppression.

## Conclusion

A strong link exists between native breeding range and susceptibility to WNV in North American owls. This relationship crosses taxonomic lines at the subfamily level and may be related to differential immunocompetence. Factors such as size and age may, to a lesser magnitude, affect exposure and susceptibility, respectively. Louse flies, common avian hematophagus parasites, may play a role in transmitting WNV; however, this role requires further examination. As WNV continues to spread, free-ranging populations of susceptible owl species may be affected.
